# Long-Term Molecular Epidemiology of *Staphylococcus epidermidis* Blood Culture Isolates from Patients with Hematological Malignancies

**DOI:** 10.1371/journal.pone.0099045

**Published:** 2014-06-04

**Authors:** Erik Ahlstrand, Bengt Hellmark, Karolina Svensson, Bo Söderquist

**Affiliations:** 1 Department of Medicine, Division of Hematology, Örebro University Hospital, Örebro, Sweden; 2 Department of Laboratory Medicine, Clinical Microbiology, Örebro University Hospital, Örebro, Sweden; 3 Faculty of Medicine and Health, Örebro University, Örebro, Sweden; Columbia University, College of Physicians and Surgeons, United States of America

## Abstract

*Staphylococcus epidermidis* is an important cause of bloodstream infections in patients with hematological malignancies. Knowledge of the long-term epidemiology of these infections is limited. We surveyed all *S. epidermidis* blood culture isolates from patients treated for hematological malignancies at the University Hospital of Örebro, Sweden from 1980 to 2009. A total of 373 *S. epidermidis* isolates were identified and multilocus sequence typing, staphylococcal chromosome cassette *mec* (SCC*mec*) typing and standard antibiotic susceptibility testing were employed to characterize these isolates. The majority of the isolates 361/373 (97%) belonged to clonal complex 2, and the 373 isolates were divided into 45 sequence types (STs); Simpson's Diversity Index was 0.56. The most prevalent STs were ST2 (243/373, 65%) and ST215 (28/373, 8%). Ninety three percent (226/243) of the ST2 isolates displayed either SCC*mec* type III or IV. ST2 and 215 were isolated during the entire study period, and together these STs caused temporal peaks in the number of positive blood cultures of *S. epidermidis*. Methicillin resistance was detected in 213/273 (78%) of all isolates. In the two predominating STs, ST2 and ST215, methicillin resistance was detected in 256/271 isolates (95%), compared with 34/100 (34%) in other STs (*p*<0.001). In conclusion, in this long-term study of patients with hematological malignancies, we demonstrate a predominance of methicillin-resistant ST2 among *S. epidermidis* blood culture isolates.

## Introduction

*Staphylococcus epidermidis* is a common colonizer of the human skin and mucous membranes that rarely causes disease in healthy individuals. However, it has been increasingly recognized as an important etiology of bloodstream infections (BSIs) in immunocompromised hosts and in patients with indwelling medical devices [1]. *S. epidermidis* is now one of the most prevalent causes of BSIs in patients with hematological malignancies [2]. The pathogenic potential of *S. epidermidis* in this population attributes to a major extent to its capacity to form biofilm, which interferes with phagocytosis and antimicrobial peptides[3] as well as reduces the efficacy of antimicrobials [4]. An additional factor in the establishment of *S. epidermidis* as a nosocomial pathogen is its widespread resistance to various antimicrobial agents. Currently, approximately 60–70% of healthcare-associated *S. epidermidis* isolates are resistant to the first-line anti-staphylococcal penicillins [5]–[7]. This resistance is mediated by the *mec*A gene that is carried by a mobile genetic element called staphylococcal cassette chromosome *mec* (SCC*mec*). Currently there are 11 different SCC*mec* structures identified in staphylococci (http://www.sccmec.org).

An essential issue is whether *S. epidermidis* BSIs are caused by nosocomial clones of *S. epidermidis* or by sporadically occurring strains from patients' commensal flora. Previous studies on this topic have demonstrated that 1) the *S. epidermidis* population is composed of hospital-associated clonal types, community-associated clonal types and clonal types that are able to survive in both environments[8], 2) *S. epidermidis* populations are highly diverse in the community [9], [10] as opposed to in clinical settings, and 3) clonal outbreaks of *S. epidermidis* can be identified in clinical settings [11]–[14]. These data are in support of that *S. epidermidis* to a major extent is a nosocomial pathogen that is spread in hospital environments.

At a local hospital level, previous reports using pulsed-field gel electrophoresis (PFGE) have demonstrated the occurrence, persistence and spread of closely related *S. epidermidis* strains [15] even during long term-follow up[16].

The improved multilocus sequence typing (MLST) scheme for *S. epidermidis* [17] is a genetic typing method based on the sequence polymorphism of fragments of seven housekeeping genes. It has proven to be highly discriminatory and can be used to elucidate relationships between strains and to identify ancestral genotypes [18]. MLST is now considered the method of choice to investigate long-term genetic relatedness within *S. epidermidis* populations [19]. Using MLST, it has been shown that the population structure of *S. epidermidis* is composed of a major and highly diverse lineage, clonal complex 2 (CC2), which can further be divided into two subdivisions or clusters: CC2-I and CC2-II [20]. MLST has been used to investigate *S. epidermidis* clonality within and between hospitals, both nationally and internationally [21]–[26]. These data indicate that a single sequence type (ST), ST2 belonging to CC2 cluster CC2-I, represents the majority of methicillin-resistant *S. epidermidis* that causes healthcare-associated infections.

In a previous study from our institution, we observed marked temporal variation in the number of positive blood cultures of *S. epidermidis* in patients with hematological malignancies, with peak incidence during the mid-1990s [27]. In this report, we analyzed all stored *S. epidermidis* blood culture isolates from patients with hematological malignancies during 30 years (from 1980 to 2009) using MLST, SCC*mec* typing, and standard antibiotic susceptibility testing. The aim was to describe the long-term molecular epidemiology of *S. epidermidis* and to elucidate whether the observed variation in incidence could be explained by the temporary occurrence of specific *S. epidermidis* genotypes.

## Materials and Methods

### Bacterial isolates

We surveyed positive blood cultures isolated from patients with hematological malignancies treated in 1980–2009 at the Division of Hematology of the Örebro University Hospital. The unit provides full hematological care except for allogeneic stem cell transplantation for the residents of Örebro County (population 285,000). After the exclusion of multiple positive cultures with the same microorganism and the same antibiogram isolated within 48 hours, we identified 567 positive blood cultures with coagulase-negative staphylococci determined by DNAse and coagulase. Of these isolates, 460 had been stored at −70°C and were possible to subculture. Coagulase-negative staphylococci were primarily determined to species level by API/ID32 Staph (BioMérieux, Marcy l'Etoile, France). In addition, nine isolates that yielded ambiguous MLST results were reevaluated to a different species level using MALDI-TOF; as a result, a total of 373 *S. epidermidis* isolates were included in the study. Fifty-five of these isolated were collected during the first, 217 during the second and 101 during the third decade of the study period.

*S. epidermidis* were isolated from 241 individual patients of whom 120 had Acute myeloid leukemia (AML), 44 Multiple myeloma, 33 Acute lymphoblastic leukemia (ALL), 18 Lymphoma, 15 Chronic lymphocytic leukemia (CLL) and 11 other hematological malignancies. Among the study isolates 234/373 (63%) grew in 2 or more blood culture sets, 120/373 (32%) grew in 1 of 2 sets, 7/373 (2%) grew in 1 of 1 set and for 10/373 (3%) data were missing. For a subset of the cohort (*S. epidermidis* isolated from 1996–2001) additional clinical data have previously been published [28]. Among these patients 83% had a central venous access and the median neutrophil count was 0.1×10^9^/L.

### Antimicrobial resistance profiles

Antibiotic susceptibility testing was performed for cefoxitin, fusidic acid, clindamycin, erythromycin, gentamicin, rifampicin, trimethoprim/sulfamethoxazole, and norfloxacin on Mueller-Hinton agar (Mueller Hinton II Agar, BD Diagnostics, Sparks, MD, USA) using the disc diffusion method (Oxoid, Cambridge, UK). Breakpoints for antibiotic resistance were according to EUCAST (http://www.eucast.org). Multidrug resistance (MDR) was defined as resistance to ≥3 antibiotic classes.

### Isolation of genomic DNA

Genomic DNA was isolated using the NorDiag Bullet with BUGS'n BEADS STI-fast-kit (DiaSorin, Dublin, Ireland). DNA preparations were stored at +4°C prior to PCR.

### Multilocus sequence typing

Amplification of the seven housekeeping genes (*arC, aroE, gtr, mutS, pyrR, tpiA*, and *yqiL*) was initially performed using the primer sequences described by Thomas et al. [17]. For isolates in which no PCR products were detected for *aroE* and *tpiA*, alternative primers described by Wang [29] and Wisplinghoff [30] were used. Fragments of the seven genes were amplified using a conventional PCR system (GeneAmp PCR System 9700, Applied Biosystem, Foster City, CA, USA). Nucleotide sequences were determined for both strands using the same primers on an ABI Prism 3130 Genetic Analyzer (Applied BioSystems, Biosystem, Foster City, CA, USA). Nucleotide sequences were compared with the MLST database via the MLST website (http://www.mlst.net) as of 1 August 2013. Individual STs were assigned to clonal complexes (CCs) using the eBURST algorithm (http://eburst.mlst.net).

### Detection of *mecA* gene

The *mecA* gene was detected using real-time PCR as previously described [31].

### SCC*mec* typing

All PCRs were run separately with specific primers. Amplification of the *ccr* gene complex (*ccr1*, *2*, *3*, and *ccrC*) and the class A *mec* complex were performed by real-time PCR using a LightCycler system (Roche Diagnostics, Mannheim, Germany) as previously described [31]. Amplification of the class B *mec* complex, and *ccr4* gene complex were performed in a conventional PCR system (GeneAmp PCR System 9700, Applied Biosystem, Foster City, CA, USA) [31]. The amplified products were identified by agarose gel (1%, High Strength Analytic Grade Agarose, Bio-Rad Laboratories, Hercules, CA, USA) electrophoresis. The *S. epidermidis* reference strain ATCC35984 was used as control. Isolates were assigned a SCC*mec* type according to the nomenclature of *S. aureus* [32]. The SCC*mec* analyses were made exclusively; first *mecA* positive isolates were analyzed for class A or B *mec* complex. Class A isolates were analyzed for *ccr3*, positive isolates were considered SSC*mec* type III and in remaining isolates no *ccr* gene complex were detected and these were considered non typeable. For class B isolates, *ccr*2 positive isolates were considered SSC*mec* IV and remaining isolates where *ccr*1 positive and considered SCC*mec* type I.

### Data analysis

The chi-squared test was used to analyze the association of categorical outcomes. Diversity among strains was evaluated using Simpson's Diversity Index, where the index equals the probability that two randomly selected strains will belong to different STs. Diversity comparisons were obtained using bootstrapping. A *p*-value of <0.05 (two-sided) was considered statistically significant.

## Results

### Multilocus sequence typing

The sequences of the seven housekeeping genes resulted in a MLST profile in 371 out of 373 isolates. For two isolates it was not possible to obtain PCR products for the *aroE* gene, even with the alternative PCR primers. These two isolates were considered untypeable by MLST (Table 1).

**Table 1:** MLST and antibiotic susceptibility results of 373 *S. epidermidis* blood culture isolates from patients with hematological malignancies.

ST	CC	N (%)	MLST profile	MRSE %	MDR %
2	2-I	243 (65)	7-1-2-2-4-1-1	95	86
215	2-II	28 (7.5)	1-6-2-1-1-16-1	93	89
73	2-II	11 (2.9)	1-5-2-6-2-1-6	0	0
23	2-II	9 (2.4)	7-1-2-1-3-3-1	89	100
5	2-II	7 (1.9)	1-1-1-2-2-1-1	57	71
22	2-I	6 (1.6)	7-1-2-2-4-7-1	100	67
38	2-II	6 (1.6)	1-2-2-5-1-1-10	33	17
57	2-II	6 (1.6)	1-1-1-1-2-1-1	0	17
59	2-II	6 (1.6)	2-1-1-1-2-1-1	67	67
6	2-II	3 (0.8)	1-1-2-2-2-1-1	67	67
25	2-II	3 (0.8)	1-6-2-1-1-1-3	100	100
295	2-II	3 (0.8)	1-1-3-6-2-1-1	0	0
17	2-II	2 (0.5)	1-1-6-2-2-1-1	0	17
19	Undefined	2 (0.5)	8-7-12-4-12-2-2	0	0
32	Singleton	2 (0.5)	1-1-7-1-3-5-14	0	0
173	2-II	2 (0.5)	1-6-6-1-2-1-10	0	100
225	2-II	2 (0.5)	1-13-7-2-2-1-29	0	0
520	2-II	2 (0.5)	1-1-2-1-1-1-33	0	0
524	2-II	2 (0.5)	1-1-2-2-4-16-1	0	0
1	2-II	1 (0.3)	1-2-2-2-1-1-10	100	100
4	2-II	1 (0.3)	1-1-6-6-2-1-1	100	100
7	2-II	1 (0.3)	1-1-1-2-4-1-1	0	0
10	2-II	1 (0.3)	1-1-1-1-3-1-1	100	100
40	2-II	1 (0.3)	1-1-2-1-3-1-1	100	100
53	365	1 (0.3)	3-1-5-5-11-4-11	0	0
85	2-II	1 (0.3)	1-1-2-2-1-1-1	0	0
86	2-II	1 (0.3)	1-2-2-1-1-1-1	0	0
88	2-II	1 (0.3)	1-1-2-1-2-1-7	0	0
136	2-II	1 (0.3)	1-1-7-2-2-1-1	0	0
152	2-II	1 (0.3)	1-1-2-6-2-1-1	0	0
190	2-II	1 (0.3)	1-1-1-2-5-1-1	0	0
210	2-II	1 (0.3)	1-1-1-2-2-1-25	0	0
218	2-II	1 (0.3)	1-1-2-6-2-16-1	0	0
327	2-II	1 (0.3)	1-1-2-1-4-1-1	0	0
337	Singleton	1 (0.3)	32-10-10-9-10-16-31	0	0
372	365	1 (0.3)	12-25-5-5-3-4-11	0	0
414	2-II	1 (0.3)	1-1-1-1-2-1-4	0	0
457	Singleton	1 (0.3)	3-16-16-5-3-19-31	0	0
521	2-II	1 (0.3)	1-23-3-6-2-1-10	0	0
522	Singleton	1 (0.3)	32-1-10-2-10-1-1	0	0
523	2-II	1 (0.3)	1-6-2-5-1-16-1	100	100
525	2-II	1 (0.3)	1-2-1-1-2-1-1	0	100
526	Singleton	1 (0.3)	8-24-17-4-12-6-9	0	0
527	2-II	1 (0.3)	43-6-6-1-2-1-10	0	100
528	2-II	1 (0.3)	1-1-7-2-2-1-29	0	100
Untypeable	-	2 (0.5)	-	50	0
Total		**373**		**78**	**73**

ST, sequence type, CC, clonal complex, MLST, multi locus sequence typing, MRSE, methicillin resistant *S. epidermidis*, MDR, multidrug resistant (≥3 antibiotic groups)

The most prevalent single ST was ST2, which constituted 243 of all 373 (65%) isolates, and this ST dominated during the entire study period. The second most prevalent ST was ST215, which comprised 28/373 (8%) of the isolates, followed by ST73 with 11/373 (3%) (Table 1).

As the majority of criteria used to determine the significance of positive blood cultures of *S. epidermidis* involve the number of positive blood culture sets of *S*. *epidermidis* [33]the predominant STs were evaluated in this respect. Among ST2 234/354 (66%), ST215 14/27 (52%) and ST73 7/11 (64%), grew in ≥2 blood culture sets. There were no significant differences comparing growth in ≥2 blood culture sets and growth in 1of 2 sets for these STs.

**Figure 1:**
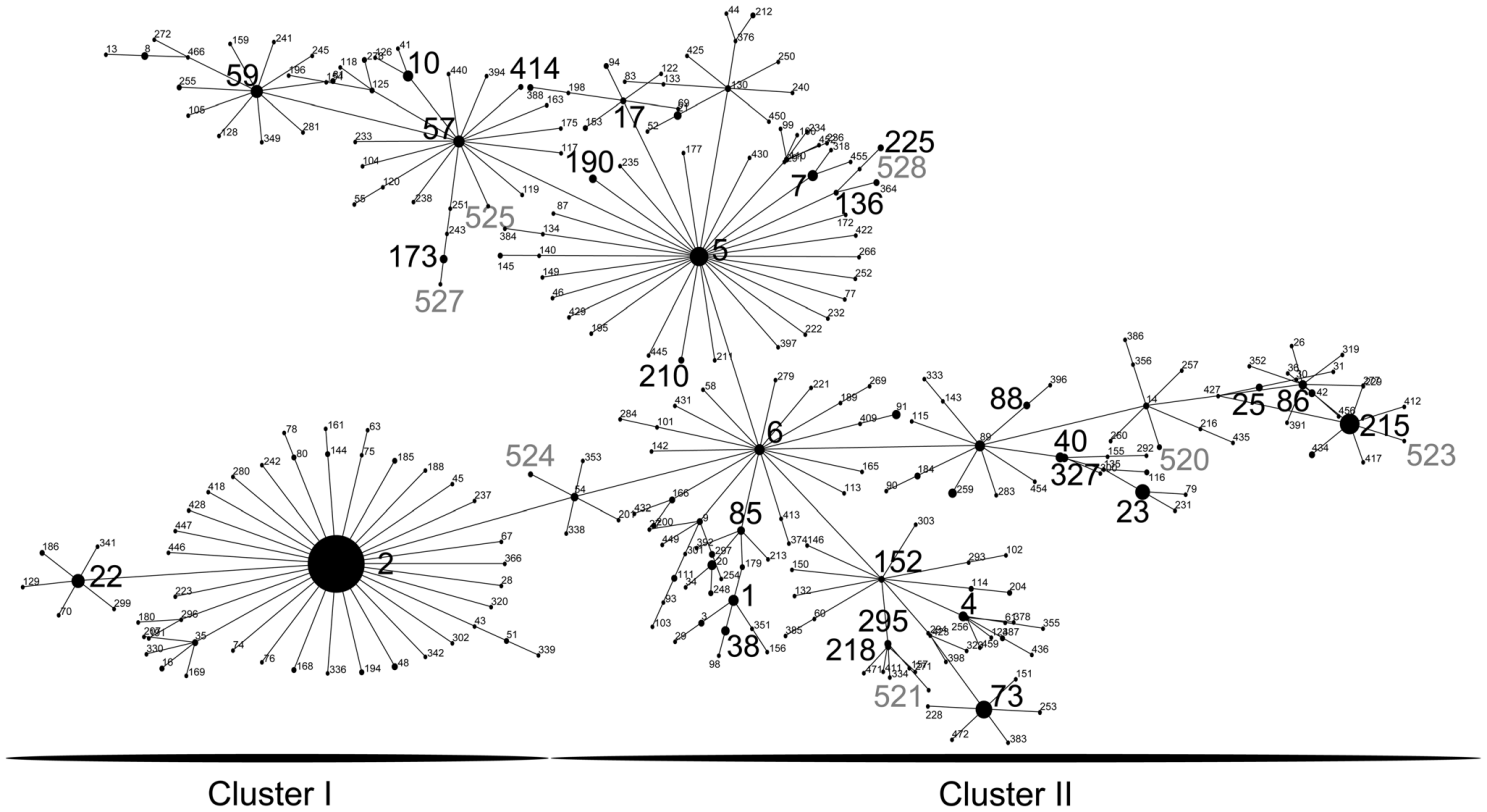
eBURST analysis of the *S. epidermidis* sequence types (STs) belonging to clonal complex 2 observed in this study compared with STs in the multilocus sequence typing (MLST) database via the MLST website (http://www.mlst.net) as of 1 August 2013. STs in large digits are STs found in this study. Not previously described STs are depicted in gray (ST520-528).

Regarding CCs, 361/373 (97%) of the isolates belonged to CC2; within CC2, 247/361 (68%) of the isolates clustered in CC2-I and 32% clustered in CC2-II (Fig. 1). Non-CC2 isolates were singletons, clustered in CC365, or in a CC without a known ST founder (Table 1). Nine STs not previously described in the MLST database were found and these were allocated ST numbers 520 to 528 in the MLST database. Of new STs, seven clustered in CC2-II (Fig. 1) and two were singletons. Simpson's Diversity Index was 0.56 for all isolates genotyped by MLST.

### SCC*mec* typing

All ST2 isolates were further characterized by SCC*mec* typing. One-hundred eighty-two isolates of 243 (75%) carried SCC*mec* type III, 44/243 (18%) SCC*mec* type IV, 9/243 (4%) were SCC*mec* type I, 2/243 (1%) were untypeable and 6/243 (2%) were *mec*A negative.

### Antibiotic susceptibility

Methicillin resistance, evaluated by cefoxitin disc diffusion, was detected in 291/373 (78%) of all isolates, and MDR was present in 272/373 (73%) isolates. There was a marked disparity in resistance profiles when comparing different STs (Table 1). In the two predominant STs, ST2 and ST215, methicillin resistance was detected in 256/271 isolates (95%), compared with 34/100 (34%) in other STs (*p*<0.001). Correspondingly, ST2 and ST215 displayed an MDR phenotype in 234/271 (86%), compared with 38/100 (38%) in other STs (*p*<0.001). Simpson's Diversity Index was 0.36 in methicillin-resistant isolates, compared with 0.94 in methicillin-sensitive isolates (*p* = 0.001).

There was a gradual increase in antibiotic resistance over time. During the first decade of the study 24/55 (44%) of the isolates were MDR and during the second and third decade 164/217 (76%) and 84/101 (83%) respectively were MDR, *p*<0.001.

### Incidence variations

**Figure 2:**
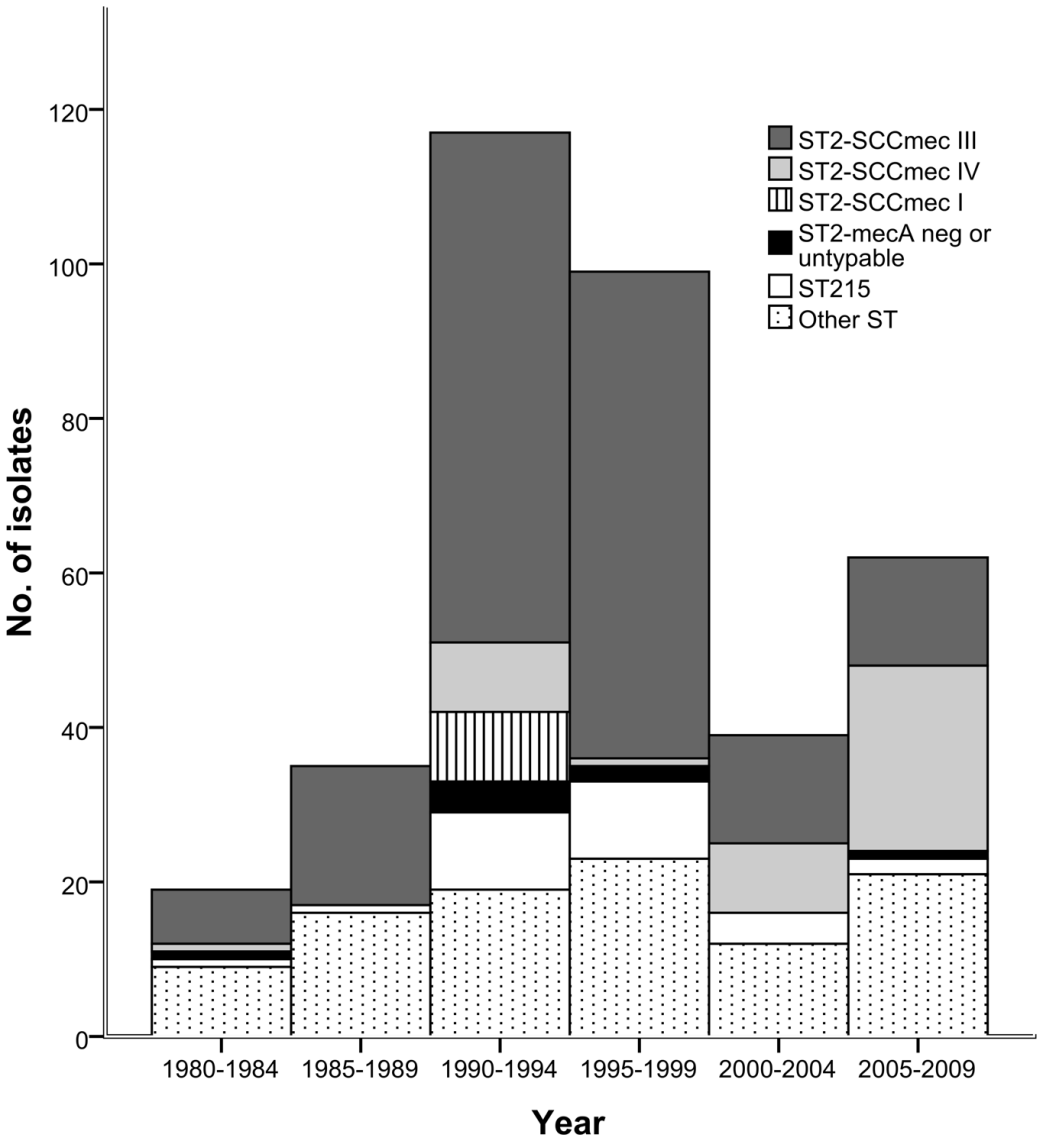
The number of positive blood cultures of S. *epidermidis* in patients with hematological malignancies. Each bar represents a 5-year period and is divided in ST2-SCC*mec* I, ST2-SCC*mec* III, ST2-SCC*mec* IV, ST2 with nontypable SCC*mec* or *mec*A negative, ST215 and other sequence types.

STs other than the two most prevalent STs (ST2 and ST215) were isolated at a relatively constant level during the study period (Fig. 2). In contrast, the number of isolated ST2 and ST215 exhibited marked variations, and together these two STs accounted for the major proportion of the peak of the number of positive blood cultures of *S. epidermidis* during the 1990s (Fig. 2).

Among ST2 isolates, SCC*mec* type III was predominating. However, during the last decade of the study period there was a trend towards increasing numbers of isolates carrying SCC*mec* type IV (Fig. 2). ST2 with SCC*mec* type I was only present during 1990–1994 (Fig. 2).

## Discussion

In the present study, we describe the molecular epidemiology of *S. epidermidis* blood culture isolates from a single hematological unit during a 30-year period. Such long-term data has not previously been presented.

The main finding is that ST2, which belongs to CC2-I, was the dominating ST of *S. epidermidis* in blood cultures at our institution during the entire study period. ST2 is a well-recognized genotype that causes nosocomial infections worldwide. It has been the most prevalent genotype reported in several studies [18], [21], [23]–[25], [34], especially in BSIs and catheter-related infections [24]. To our knowledge only one study reported ST5 more common than ST2 [26]. ST2 has only rarely been described to colonize healthy individuals in Swedish studies [9], [35], and ST2 was not found at all in Chinese commensal isolates [25]. Furthermore, ST2 has been associated with many of the currently known virulence factors, such as biofilm production and antibiotic resistance [24], [25], which is in accordance with the association between ST and methicillin resistance, as well as the MDR demonstrated in this study. However, no single virulence factor has been linked to the success of ST2 as a nosocomial pathogen.

In our study almost all (93%) ST2 isolates carried either SCC*mec* type III or IV, a finding that is in accordance with previous studies in the field [24], [25], [36].

The second most prevalent ST in this study, ST215, has previously been described as the cause of a wide range of healthcare-associated infections in Sweden and Norway [22], [35]. Interestingly, ST215 has not been described among commensal isolates [9], [35].

ST73 was the third most isolated ST. ST73 has been described as a colonizer of healthy subjects in Sweden and Portugal [9], [18], which is consistent with the antibiotic-sensitive phenotype of ST73 in this study.

Although the investigated *S. epidermidis* isolates were either causative agents of bacteremia or contaminants, the genetic diversity according to STs was considerably lower than in global [18] and multicenter studies [26]. In contrast, a Finnish study found ST2 alone among 60 *S. epidermidis* isolates from bacteremic patients in three units at the same hospital [23]. These differences underscore that *S. epidermidis* infections in general are hospital acquired and caused by genetically related strains that persist in the hospital environment (i.e., endemic clones).

**Figure 3:**
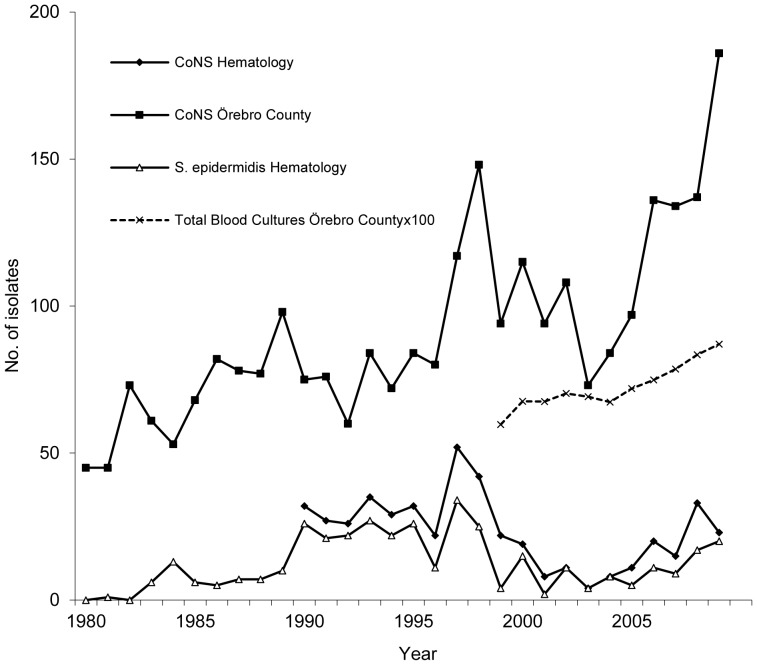
The number of positive blood cultures of coagulase-negative staphylococci (CoNS) in Örebro County, Sweden (filled squares); CoNS at the Division of Hematology, Örebro University Hospital (filled diamonds); and *S. epidermidis* in patients with hematological malignancies, Örebro University Hospital (study population, open triangles). The total number of blood cultures performed in Örebro County from 1999 to 2009 is also shown (crosses).

In our institution, we observed a marked variation in the number of positive blood cultures of *S. epidermidis* during 1980–2009 (Fig. 3). This variation corresponded to a variation in the total number of positive blood cultures of coagulase-negative staphylococci in the entire county of Örebro (Fig. 3). We have no plausible explanation for this phenomenon since we do not know of any significant changes in the patient population or in patient care (e.g., antimicrobial prophylaxis policies and blood culture methodology) that could have influenced these fluctuations. Our data do not support the hypothesis that these variations are caused by transient occurring MLST genotypes, because the main part of the incidence peaks can be explained by increased number of positive cultures of the predominating STs, ST2 and ST215. We report temporal variations of different SCC*mec* types of ST2 isolates during the study period. During 1990–1994 ST2 carrying SCC*mec* type I were temporarily isolated and during the last decade of the study there was a gradual increase in the number of ST2 carrying SCC*mec* type IV. Interestingly, this increase coincides with the emergence of community acquired methicillin resistant *S. aureus* (MRSA) even though the study was performed in an area with a low incidence of MRSA, (approximately 1% of clinical isolates). In the time period with the higher incidence of *S. epidermidis* during the 1990ies isolates of ST2 carrying SCC*mec* type III predominate which may indicate that SCC*mec* type III provides a fitness advantage in the hospital environment. In summary, these observations may reflect that health care-associated *S. epidermidis* infections at least partly are caused by temporally occurring genotypes.

One strength of the present report is the restriction of the study population to a limited geographic area with solely one microbiological laboratory service. Thereby it was possible to include the vast majority of *S. epidermidis* blood culture isolates from patients with hematological malignancies. However, a limitation is that *S. epidermidis* isolates that were not stored at the time of diagnosis had to be excluded. Isolates not stored predominantly represented multiple positive cultures from the same infection episode or isolates with growth in only one blood culture set that were interpreted as contamination at the time of diagnosis. Few isolates were identified during the 1980s, possibly because less intensive chemotherapy schedules were used in 1980s, but it might also indicate a selection bias in that a smaller proportion of *S. epidermidis* isolates were stored during this time. Another limitation is that it was not possible to assess whether the analyzed *S. epidermidis* isolates had caused true BSIs or were blood culture contaminants. However, such distinction is not easily made since a uniform gold standard to make this important distinction is lacking. The present study population represents a high risk population for *S. epidermidis* BSIs and most likely a great proportion of the included isolates represented true BSIs. PFGE may have provided additional discriminatory power as well as a different SCC*mec* typing strategy allowing the identification of potential multiple SCC*mec* types.

In conclusion, although *S. epidermidis* is known to possess a highly dynamic and plastic genome, MLST has made it possible to investigate the distribution of various genotypes at a single unit during a 30-year period, which underscores the importance of MLST in studying the long-term relatedness of *S. epidermidis* infections. The predominant STs of *S. epidermidis* blood culture isolates obtained from patients with hematological malignancies from 1980 to 2009 were ST2 and ST215. The findings in the study add to the comprehension that healthcare-associated blood culture isolates of *S. epidermidis* in immunocompromised patients belong to the same genetic background.
